# Randomized double-blind safety comparison of intravenous iron dextran versus iron sucrose in an adult non-hemodialysis outpatient population: A feasibility study

**DOI:** 10.1186/s12878-016-0046-8

**Published:** 2016-03-11

**Authors:** Martha L. Louzada, Cyrus C. Hsia, Fatimah Al-Ani, Fiona Ralley, Anargyros Xenocostas, Janet Martin, Sarah E. Connelly, Ian H. Chin-Yee, Leonard Minuk, Alejandro Lazo-Langner

**Affiliations:** Department of Medicine, Division of Hematology, London, ON Canada; University of Western Ontario, London, ON Canada; Department of Anesthesia and Perioperative Medicine, London, ON Canada; Department of Pharmacy, London Health Sciences Centre, London, ON Canada; Department of Epidemiology & Biostatistics, London, ON Canada; London Health Sciences Centre, Department of Medicine, Division of Hematology. Rm E6-219A, Victoria Hospital, 800 Commissioners Road E., London, ON N6A 5W9 Canada

**Keywords:** Intravenous iron, Iron dextran, Iron sucrose, Safety, Feasibility, Randomized controlled trial

## Abstract

**Background:**

Intravenous iron therapy is a treatment option for iron deficient patients who are intolerant to oral iron or where oral iron is ineffective, but with possible adverse effects. Currently, prospective studies comparing different intravenous iron formulations are needed to determine safety and efficacy of these agents.

**Methods:**

We conducted a prospective, double-blind, randomized controlled trial (RCT) to assess the feasibility of a trial comparing the safety of high molecular weight intravenous iron dextran, Infufer®, with intravenous iron sucrose, Venofer®, in non-hemodialysis adult outpatients. Primary outcome was the occurrence of immediate severe drug reactions.

**Results:**

We enrolled 143 patients in a one-year period. Overall, 45/143 (31.5 %) patients (20 iron dextran, 25 iron sucrose) developed 48 infusion reactions (14 immediate, 28 delayed, and 3 both). The risk of an immediate reaction was similar in both groups, 9/73 (12.3 %) iron dextran versus 8/70 (11.4 %) iron sucrose, RR = 0.93 (95 % CI; 0.38 to 2.27). The risk of a delayed reaction was significantly higher in the iron sucrose group 22/70 (31.4 %) versus the iron dextran group 9/73 (12.3 %), RR = 2.55 (95 % CI; 1.26 to 5.15; *p* = 0.0078).

**Conclusion:**

In this limited feasibility study, no major differences in immediate reactions were seen, but a significantly higher number of delayed reactions were seen in the iron sucrose group. Further, under our assumptions and design a full RCT to evaluate the safety of different intravenous iron preparations is not feasible. Future studies should consider modifying the clinical outcomes, utilize multiple centers, and consider other emerging parenteral iron formulations. (ClinicalTrials.gov NCT005936197 January 3, 2008).

## Background

Iron deficiency is the most common cause of anemia worldwide affecting up to 50 % of children under 5 years of age and up to 20 % of women under the age of 50 [1–3]. Use of oral iron supplementation is the standard first line treatment; however, it is associated with several side effects that may lead to lack of compliance. Adverse drug reactions (ADRs) to oral iron can be as high as 70 % with associated non-adherence rates of 70 % [4]. Intravenous iron may be an alternative for patients intolerant or unresponsive to oral iron formulations and is most widely used in patients with chronic kidney disease on hemodialysis [5–8]. It is also frequently used preoperatively with or without erythropoietin to augment hemoglobin levels prior to surgery, in patients with iron deficiency anemia secondary to gastrointestinal bleeding or pre-dialysis patients with chronic kidney disease where oral iron supplementation is insufficient [5, 9].

In the non-hemodialysis setting there is evidence to support the effectiveness of intravenous iron but relatively little evidence comparing the safety of different intravenous iron formulations. A few observational studies and randomized trials comparing the adverse reactions of iron dextran to iron sucrose have suggested the development of more frequent and serious ADRs in patients using iron dextran [9–15]. A previous retrospective study conducted at our centre showed higher incidence rates of adverse events and severe reactions in patients receiving iron dextran compared to iron sucrose [16]. However, these findings are difficult to interpret because different doses of intravenous iron have been used in studies and different formulations exist for the same agent (e.g., high versus low molecular weight iron dextran).

Due to extensive intravenous iron utilization in hospitals, the need of prospective randomized trials to inform clinical decisions in selection of appropriate intravenous iron formulations is increasing. In this study, we sought to evaluate the feasibility of a randomized trial to compare the safety of a high molecular weight iron dextran with iron sucrose in non-hemodialysis adult outpatients.

## Methods

### Participants

The study was conducted at the London Health Sciences Centre, a University-affiliated academic center in London, Ontario Canada. We included adult (18 years of age or older (outpatients with iron deficiency anemia eligible to receive intravenous iron as a part of their clinical management. Iron deficiency anemia was defined as hemoglobin less than 130 g/L and a ferritin of less than 50 μg/L. This hemoglobin level was chosen because our perioperative blood conservation program identifies potential surgical patients with hemoglobin values between 100 and 130 g/L for possible intravenous iron to reduce the exposure to allogeneic blood products. Patients were excluded if they were on hemodialysis, had previous exposure to any form of intravenous iron or were unable to provide written informed consent.

### Study design and sample size

The primary objective of this study was to assess the feasibility and to inform details for the design of a future randomized controlled trial to be conducted at our centre comparing the safety of equal doses of intravenous iron dextran or iron sucrose in non-hemodialysis adult patients. Based on the results of our previous retrospective study of adverse reactions to intravenous iron [16], we calculated that we would need to enrol 213 patients per group to demonstrate a 5 % difference between groups for the main outcome at the 95 % level of significance with a power of 80 %. In order for such a trial to be considered feasible we would need to enrol approximately 100 patients per year. Based on our clinical volumes we anticipated that we could identify 120 potential patients per year. If 90 % agreed to participate, then we would be able to complete accrual for the full trial within 4 years.

The study was designed as a double-blinded randomized controlled trial comparing equal doses of intravenous iron dextran with intravenous iron sucrose. Randomization sequences were computer-generated via a third party (IBM, San Jose, California, USA) and stratified by site (2 sites), in blocks of 8. Randomization tables were only accessible by our central pharmacy requiring this information for concealment of iron products. Participants could choose to stop the study any point during the study or be unblinded at the discretion of the treating physician if it was felt that continuing would harm the patient. Subsequently, cross-over to the other agent could be done at the discretion of the treating physician. The study complied with the Declaration of Helsinki, Health Canada and the international conference on harmonization – good clinical practice (ICH-GCP) guidelines. The study protocol was approved by the Research Ethics Board of the University of Western Ontario (HSREB 13767). Written informed consent was obtained from all participants. This study was registered at ClinicalTrials.gov with number NCT00593619 January 8, 2008.

### Interventions

Patients were randomized to receive either intravenous iron dextran with an estimated molecular weight of 200 kDa (Infufer®, Sandoz Canada Inc., Montreal, Canada) or iron sucrose (Venofer®, Luitpold Pharmaceuticals Inc., Shirley, New York, USA) at a dose of 300 mg given in 250 mL of normal saline and administered over 2 h with the first 25 mg over 10 min as a test dose. Each study drug was concealed and had a unique study label. No pre-medications were permitted. Before and after iron infusion, samples were obtained for complete blood count and serum ferritin.

### Study outcomes

**Fig. 1:**
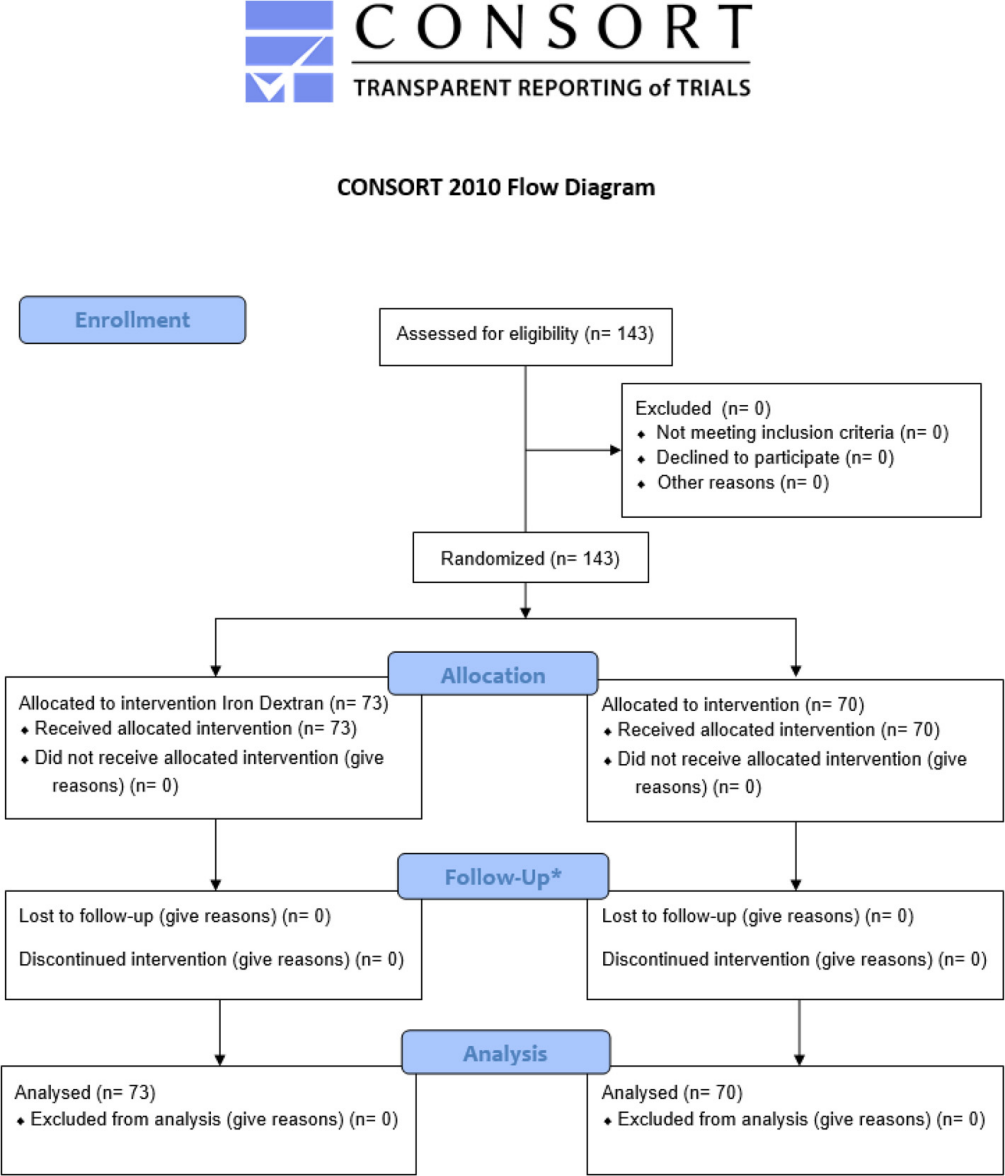
Study flow diagram. *Reaction assessment performed during infusion (immediate reaction) and at 24 h post infusion

The primary feasibility outcome of the study was enrollment of at least 100 patients per year. The primary clinical outcome of the study was the occurrence of immediate severe adverse reactions (ADRs). Secondary outcomes were the occurrence of: immediate and delayed serious ADRs; immediate anaphylactic/anaphylactoid ADRs, immediate combined mild and moderate ADRs, delayed ADRs, all-cause mortality; mean time physicians spent managing ADRs; mean time nurses spent managing ADRs; and absolute difference in hemoglobin, platelet and ferritin. We also planned to collect costing data for a cost effectiveness analysis. ADRs were recorded including the onset (in minutes from initial administration), duration and description of symptoms/signs, intervention(s) applied, and any additional nursing time required to manage the reaction. ADRs were considered immediate if they occurred during the infusion time or delayed if occurred within the first 24 h post-infusion (Fig. 1). All patients were contacted at home via telephone after 24 h by a member of the research team for assessment of delayed reactions. The severity of ADRs was classified according to the National Cancer Institute (NCI) Common Terminology Criteria for Adverse Events v3 · 0 guidelines: (Table 2). Three blinded assessors (2 Hematologists and 1 Cardiologist), blinded to patient allocation, reviewed the ADRs and independently adjudicated the type and severity of ADRs. Discrepancies were resolved by consensus with a fourth investigator.

### Statistical analysis

All statistical analyses were performed on an intention-to-treat basis. Baseline characteristics of participants, primary and secondary outcomes were analyzed by means of descriptive statistics. For comparison between groups we used Fisher’s exact test for categorical variables and unpaired t-test for continuous variables. We calculated relative risk (RR) and 95 % confidence intervals (95 % CI) for primary and secondary outcomes, using the iron sucrose group as reference. *P*-values <0.05 were considered statistically significant.

## Results

Between January 2008 and January 2009 we enrolled 143 patients. The study was terminated early after an interim analysis found four severe ADRs occurred. All potentially eligible patients were approached and agreed to participate in the study (Fig. 1). No participants withdrew consent or were lost to follow-up. Patient characteristics are shown in Table 1. Of the participants, 46/143 (32.2 %) were males, the median age was 68 years (standard deviation 17.6) and the most frequent indication for intravenous iron therapy was pre-operative iron supplementation in 117/143 (81.8 %).

**Table 1:** Baseline characteristics of included patients

Characteristics	Iron dextran	Iron sucrose	*P*
	*N* = 73	*N* = 70	
Female sex (%)	45 (61.6)	52 (74.3)	0.112
Age, years (sd)	70 (17.6)	66 (17.4)	0.872
IV iron indication [n(%)]			
Pre-operative			
Cardiovascular surgery	18 (25)	14 (20)	
Gastrointestinal surgery	10 (14)	8 (11)	
Orthopedic surgery	32 (44)	36 (51)	
Other surgeries	1 (1)	1 (1)	
Bleeding			
Menorrhagia	2 (3)	2 (3)	
Acute GI bleed	4 (5)	5 (5)	
Chronic GI Bleed	3 (5)	1 (3)	
Other			
Malignancy	2 (3)	2 (3)	
Indication not available	1 (2)	1 (3)	
Other characteristics [n(%)]			
Chronic kidney disease	7 (10)	2 (3)	0.167
Previous treatment [n(%)] (available data)	68	57	
PO iron	53 (73)	50 (71)	
IM iron	3 (4)	0 (0)	
Erythropoietin (EPO)	2 (3)	1 (2)	
Both iron & EPO	33 (45)	30 (43)	
None	10 (14)	6 (4)	

*Abbreviations*: *GI* gastrointestinal, *IM* intramuscular Iron, *PO* oral, *sd* standard deviation

**Fig. 2:**
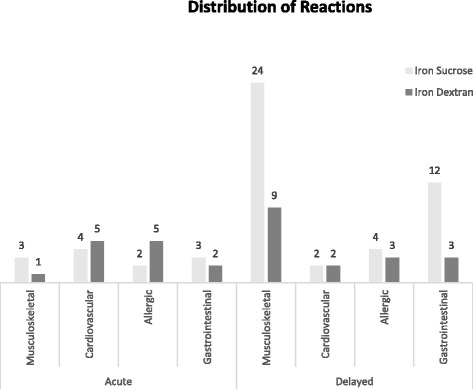
Distribution of reactions

Overall, 45/143 (31.5 %) patients developed 48 infusion reactions (14 immediate, 28 delayed, and 3 with both). The risk of an immediate reaction was similar in both groups: 9/73 (12.3 %) iron dextran and 8/70 (11.4 %) iron sucrose (RR = 0.93, 95 % CI; 0.38 to 2.27, *p* = 0.873). However, the risk of a delayed reaction was significantly higher in the iron sucrose group [22/70 (31.4 %)] versus 9/73 (12.3 %) in the iron dextran group (RR = 2.55, 95 % CI; 1.26 to 5.15; *p* = 0.0078).A detailed list of immediate and delayed reactions are provided in Table 2. The reactions were classified into four major categories (musculoskeletal, cardiovascular, allergic, and gastrointestinal) in Fig. 2 and highlight that there were more musculoskeletal and gastrointestinal delayed adverse reactions with iron sucrose.

**Table 2:** Adverse drug reactions and severity classified according to the National Cancer Institute Common Toxicity Criteria

	Sex	Iron formulation	Reaction (Grade of severity)
Immediate reactions			
1.	F	Sucrose	Back pain (2), tachycardia (1), nausea (1)
2.	F	Sucrose	^a^Allergic Reaction (3)
3.	F	Dextran	Chest pain (2)
4.	F	Dextran	Pruritus (1)
5.	F	Sucrose	Taste alteration (1)
6.	M	Sucrose	Headache (2), Tachycardia (1)
7.	F	Dextran	Dyspnea (1), Flushed (1), Abdominal distension/bloating (3)
8.	F	Dextran	^a^Urticaria (3)
9.	F	Sucrose	Abdominal distension/bloating (1)
10.	M	Dextran	Dyspnea (2)
11.	F	Sucrose	Urticaria (2)
12.	F	Sucrose	Flushing (2)
13.	F	Sucrose	^a^Hypotension (3), back pain (2)
14.	F	Dextran	Hypotension (2), Urticaria (2), Chills (1)
15.	F	Dextran	Nausea (1)
16.	F	Dextran	^a^Allergic Reaction (3)
17.	F	Dextran	Neuropathy - sensory (2)
Delayed reactions			
1.	M	Sucrose	Arthralgia (1), Myalgia (1)
2.	F	Dextran	Nausea (1), Headache (1), Chills (1), Abdominal distension (1)
3.	F	Sucrose	Fatigue (1)
4.	F	Dextran	Headache (1), Fatigue (1)
5.	F	Dextran	Presyncope (2)
6.	F	Sucrose	Arthralgia (1), Myalgia (1), Chills (1)
7.	F	Sucrose	Diarrhea (1), Abdominal distension (1), Headache (2)
8.	F	Dextran	Headache (1)
9.	F	Sucrose	Headache (2), flushes (1)
10.	F	Dextran	Pruritus (1)
11.	F	Sucrose	Urticaria (2), Fever (2), Presyncope (2)
12.	M	Dextran	Back pain (2)
13.	F	Sucrose	Fatigue (1), Arthralgia (1)
14.	M	Sucrose	Headache (1), Abdominal distension (1)
15.	F	Sucrose	Abdominal distension (1), Fever (1)
16.	F	Sucrose	Fever (1), Headache (2)
17.	F	Sucrose	Diarrhea (1)
18.	F	Sucrose	Headache (1), Back pain (1)
19.	F	Sucrose	Pruritus (1)
20.	F	Dextran	Urticaria (2)
21.	F	Sucrose	Urticaria (1)
22.	F	Sucrose	Edema limbs (1)
23.	F	Sucrose	Headache (1)
24.	F	Sucrose	Abdominal distension (1), Diarrhea (1)
25.	F	Sucrose	Abdominal distension (1)
26.	F	Dextran	Arthralgia (1), Myalgia (1), Back pain (1)
27.	F	Sucrose	Chills (1), Generalized muscle weakness (1), Nausea (1)
28.	M	Sucrose	Nausea (1), Headache (1), Abdominal pain (1)
29.	F	Dextran	Nausea (1), Headache (1), Presyncope (2)
30.	F	Sucrose	Nausea (1)
31.	F	Sucrose	Arthralgia (1), Myalgia (1), Chills (1)

^a^Patients who required further intervention including transfer to emergency department

Severity of events according to the National Cancer Institute Common Toxicity Criteria

0 = No adverse event or within normal limits

1 = Mild adverse event

2 = Moderate adverse event

3 = Severe and undesirable adverse event

4 = Life-threatening or disabling adverse event

5 = Death related to adverse event

After infusion start, the mean time for the occurrence of immediate reactions was 32 min (range 2 to 120). All of the immediate and delayed reactions were transient and self-limited with deaths reported. There were nine immediate reactions (6 iron dextran and 3 iron sucrose) that occurred within the first 10 min that can be considered reactions within the “test dose” period of time. Eighteen patients required medical intervention with no significant difference between the dextran or sucrose arms. While most of the ADRs were mild in severity (Grade 1 or 2), a total of four patients (2.7 %) were considered to have severe grade 3 or 4 ADRs and were sent to the emergency department for appropriate management, two in each study arm, and were unblinded at the request of the treating physicians. A Data Safety Monitoring committee stopped the trial due to these events. Only one patient was crossed over to the other product (from iron sucrose to iron dextran) at the discretion of the treating physician without any further complications.

Hematologic parameters including hemoglobin, platelet count and ferritin were evaluated before and after the first intravenous iron infusion. There were no significant differences between groups (data not shown). Finally, although we planned to estimate resource utilization and costs, unfortunately this data was not accurately recorded and therefore it is not reported.

## Discussion

We conducted a study to evaluate the feasibility of a randomized controlled trial comparing the safety of two parenteral iron formulations in previously untreated non-dialysis iron deficient patients. The study was stopped prematurely due to the occurrence of 4 severe ADRs requiring physician assessment and intervention. Although we were able to exceed our recruitment target with a total of 143 patients enrolled in a one-year period, with the observed rate of severe immediate reactions, a randomized trial designed to detect a 2 % difference in immediate ADRs would not be feasible as a single centre study. The design of the study with a one point in time evaluation and a short follow up that did not require extra hospital visits or blood tests were attractive features that maximized patients’ participation.

With respect to the clinical outcomes of the study, we found no significant difference in the incidence of total or immediate ADRs between iron dextran group and iron sucrose group. However, the risk of a delayed reaction was significantly higher in the iron sucrose group. The incidence of the overall number of reactions is much higher than previously reported. Our data is congruent with previously published available literature with respect to the incidence of severe adverse reactions of high molecular weight iron dextran and iron sucrose. Overall, studies have reported extremely low rates of serious adverse reactions with different preparations of intravenous iron. In particular, anaphylactoid reactions and death are extremely rare. Studies performed before 2000, using high molecular weight dextran suggest an incidence of severe ADRs of about 1 % [11, 13, 17]. A small study compared the safety of low molecular weight iron dextran with iron sucrose in patients with chronic kidney disease and showed that the incidence of side effects associated with iron-dextran was not different than that of iron-sucrose [18].

A recent single institution retrospective study of 619 unique patients showed that no serious ADRs were associated with intravenous iron use in patients receiving low molecular weight iron dextran, iron sucrose, ferric gluconate and high molecular weight dextran. Regarding the incidence of ADRs, low molecular weight dextran and ferric gluconate were similar and both caused less ADRs than iron sucrose. High molecular weight dextran, although used in a small number of patients (only nine patients in that study), was associated with a high rate of ADRs (44.4 %) [19] and other studies suggest that high molecular weight iron dextran formulation has a higher incidence of adverse outcomes compared to iron sucrose [6, 7, 10, 20, 21].

Whereas in our earlier retrospective study, we found that the risk of severe ADRs was 7-fold higher with Infufer® compared to Venofer® [16], in our current study, we were not able to show a difference. We found similar incidence rates of acute reactions and severe ADRs, but a surprising significant increase in delayed ADRs in the sucrose group (*p* = 0.078). Of particular note, when reactions were categorized there appeared to be more musculoskeletal and gastrointestinal delayed reactions with iron sucrose. However, we cannot completely rule out that the lower incidence of delayed ADRs in the iron sucrose group in our previous study may have been due to reporting bias. Further, the majority of our patients, 81 to 83 %, received intravenous iron in the pre-operative setting limiting the generalizability of this data to patients with iron deficiency in general routine practice.

In this study, we did not aim to collect markers of oxidative stress or other markers to determine the mechanisms of these reactions. In addition to our study’s early termination another limitation of our study is that we were not able to evaluate the risk of adverse reactions with subsequent intravenous iron infusions. Nevertheless, studies suggest that up to 70 % of ADRs occur during or right after the first intravenous iron infusion [11–13]. Further, we used iron sucrose at a dilution of 1.2 mg/mL (a lower end of dilution for this product) and dilution of nanoparticle colloidal suspensions such as intravenous iron formulations leads to reduced stability due to ionic shielding. However, both products were administered using the same dilution at our commonly used iron sucrose concentration of 1.2 mg/mL (that was also used in our previous study).

## Conclusion

We conducted a randomized controlled trial (RCT) to evaluate the feasibility for accrual and to inform the design of a future trial comparing the safety of intravenous iron dextran versus iron sucrose in non-hemodialysis adult patients at a single center. Whereas accrual was possible under our assumptions in the first year, it was stopped early. With the limited data, we found no significant difference in the incidence of immediate ADRs and a rate of delayed reactions that was significantly higher in the sucrose group. Given these findings we conclude that under our assumptions and design that a full RCT is not feasible to be conducted at a single center. Future studies should consider modifying the clinical outcomes, utilize multiple centers, and consider other emerging parenteral iron formulations.

## Abbreviations

ADR: adverse drug reaction
ICH-GCP: international conference on harmonization – good clinical practice
NCI: National Cancer Institute
RCT: randomized controlled trial
RR: relative risk
